# Optimizing Gaussian process regression (GPR) hyperparameters with three metaheuristic algorithms for viscosity prediction of suspensions containing microencapsulated PCMs

**DOI:** 10.1038/s41598-024-71027-9

**Published:** 2024-08-31

**Authors:** Tao Hai, Ali Basem, As’ad Alizadeh, Kamal Sharma, Dheyaa J. jasim, Husam Rajab, Mohsen Ahmed, Murizah Kassim, Narinderjit Singh Sawaran Singh, Hamid Maleki

**Affiliations:** 1 Key Laboratory of Advanced Manufacturing Technology, Ministry of Education, Guizhou University, Guiyang, 550025 China; 2 School of Computer and Information, Qiannan Normal University for Nationalities, Duyun, 558000 Guizhou China; 3 Faculty of Data Science and Information Technology, INTI International University, 71800 Nilai, Malaysia; 4 Faculty of Engineering, Warith Al-Anbiyaa University, Karbala, 56001 Iraq; 5 Department of Civil Engineering, College of Engineering, Cihan University-Erbil, Erbil, Iraq; 6 Institute of Engineering and Technology, GLA University, Mathura, U.P. 281406 India; 7 Department of Petroleum Engineering, Al-Amarah University College, Maysan, Iraq; 8 College of Engineering, Mechanical Engineering Department, Alasala University, King Fahad Bin Abdulaziz Rd., Amanah, P.O.Box: 12666, 31483 Dammam, Kingdom of Saudi Arabia; 9 Imam Abdulrahman Bin Faisal University, P.O. Box 1982, Dammam, 31441 Eastern Province Kingdom of Saudi Arabia; 10 Institute for Big Data Analytics and Artificial Intelligence (IBDAAI), Universiti Teknologi MARA, 40450 Shah Alam, Selangor Malaysia; 11 School of Electrical Engineering, College of Engineering, Universiti Teknologi MARA, 40450 Shah Alam, Selangor Malaysia; 12 Department of Mechanical Engineering, Isfahan University of Technology, Isfahan, Iran; 13 Artificial Intelligence Research Center (AIRC), Ajman University, P.O. Box 346, Ajman, UAE

**Keywords:** Microencapsulated PCM, Thermal energy storage, Gaussian process regression, Genetic algorithm, Particle swarm optimization, Marine predators algorithm, Energy science and technology, Engineering, Materials science, Nanoscience and technology

## Abstract

Suspensions containing microencapsulated phase change materials (MPCMs) play a crucial role in thermal energy storage (TES) systems and have applications in building materials, textiles, and cooling systems. This study focuses on accurately predicting the dynamic viscosity, a critical thermophysical property, of suspensions containing MPCMs and MXene particles using Gaussian process regression (GPR). Twelve hyperparameters (HPs) of GPR are analyzed separately and classified into three groups based on their importance. Three metaheuristic algorithms, namely genetic algorithm (GA), particle swarm optimization (PSO), and marine predators algorithm (MPA), are employed to optimize HPs. Optimizing the four most significant hyperparameters (covariance function, basis function, standardization, and sigma) within the first group using any of the three metaheuristic algorithms resulted in excellent outcomes. All algorithms achieved a reasonable R-value (0.9983), demonstrating their effectiveness in this context. The second group explored the impact of including additional, moderate-significant HPs, such as the fit method, predict method and optimizer. While the resulting models showed some improvement over the first group, the PSO-based model within this group exhibited the most noteworthy enhancement, achieving a higher R-value (0.99834). Finally, the third group was analyzed to examine the potential interactions between all twelve HPs. This comprehensive approach, employing the GA, yielded an optimized GPR model with the highest level of target compliance, reflected by an impressive R-value of 0.999224. The developed models are a cost-effective and efficient solution to reduce laboratory costs for various systems, from TES to thermal management.

## Introduction

Thermal energy storage (TES) is a captivating subject of academic inquiry that centers on capturing and retaining thermal energy for subsequent utilization in various applications. Its significance stems from its capacity to effectively address challenges related to energy demand management, grid stability, and the seamless integration of renewable energy sources^1^. TES is pivotal in promoting efficient energy utilization, diminishing dependence on fossil fuels, and fostering sustainability^2^. TES systems operate by amassing surplus thermal energy during periods of low demand or excessive production. This stored energy can then be effectively retrieved and harnessed during peak demand periods or when renewable energy sources experience temporary unavailability. The applications of TES are inherently diverse and transcend multiple sectors. Within the building industry, TES systems can be seamlessly integrated to regulate indoor temperatures, optimize energy consumption, and alleviate the strain on HVAC systems^3^. TES facilitates enhanced efficiency in industrial processes, such as power plants, by stockpiling excess heat for subsequent conversion into electricity or process heat^4^. Moreover, TES has extensive applications in solar thermal power plants, guaranteeing uninterrupted power generation by accumulating surplus solar energy during daylight hours for utilization during nighttime^5^.

Latent heat thermal energy storage (LHTES) is a significant approach within the TES field, which harnesses the potential of phase change materials (PCMs) to store and release thermal energy during phase transitions^6,7^. PCMs can absorb or release substantial amounts of energy as latent heat when transitioning between different phases, such as solid to liquid or liquid to gas^8^. To enhance the stability and containment of PCMs, researchers have developed microencapsulated phase change materials (MPCMs), wherein the PCM is encapsulated within minuscule containers^9^. This innovative approach offers several advantages, including reduced leakage of PCMs, improved heat transfer characteristics, and enhanced compatibility across various applications^10^. MPCMs have found significant utility in diverse areas, such as solar energy storage, building heating and cooling systems, and waste heat recovery^11^. Their integration into TES technologies drives progress in the field and contributes to the broader objective of advancing sustainable energy utilization.

The thermo-physical properties (TPPs) of MPCMs and MPCM-based solutions hold significant importance in TES applications. TPPs directly impact the heat transfer efficiency, energy storage capacity, and stability of the MPCM suspensions. Researchers have investigated the TPPs of MPCMs and MPCM-based solutions in recent years. Sarı et al.^12^ developed microencapsulated heptadecane in a CaCO_3_ shell to improve thermal conductivity (TC) and prevent leakage during the phase change process. Their MPCM exhibited good chemical stability, dependability, and thermal degradation durability. They found that with enhanced TC, the microcapsules have the potential for various TES applications, especially air conditioning, textile management, and food preservation. In a recent study by Liu et al.^13^, a novel MPCM with improved TC was developed. The MPCM was fabricated using an n-eicosane core and a phenol–formaldehyde resin shell, revised with nanomaterials (NMs). The researchers observed a significant enhancement in TC with the addition of NMs, particularly in the case of boron nitride-based MPCM (61%) and silicon carbide-based MPCM (97%). This study highlights the potential of NMs in enhancing the TPPs of MPCMs. Dutkowski and Kruzel^14^ performed experimental investigations on the TC of water-based slurry consisting of MPCM and water-propylene glycol solutions. Their findings revealed that elevating the slurry temperature increased TC for solid and liquid PCM states. Xia et al.^15^ conducted a study to synthesize a novel type of MPCM with a melamine–formaldehyde shell reinforced by boron nitride (BN) to enhance TC. The microcapsules exhibited desirable phase change conditions and demonstrated privileged thermal cycling stability. TC analysis revealed that incorporating BN into the microcapsule shell resulted in notable improvements in the TC of the MPCM.

Liu and Zhou^16^ numerically investigated the flow and heat transfer features of MPCM suspension in a microchannel. The study analyzed the impact of Reynolds numbers, mass fractions (MFs), and MPCM size distributions on the system's behavior. The findings revealed that an increase in MPCM MF by up to 30% led to a transition from Newtonian to pseudoplastic behavior. Moreover, as the MPCM particle size increased within the range of 0.1 to 10 μm, the non-Newtonian behavior of the suspension became more pronounced. Srinivasaraonaik et al.^17^ examined the effects of different dosages of MPCM on cement paste specimens on thermal comfort. Their findings indicated that the incorporation of MPCM decreased compressive strength, bulk density, and TC while increasing porosity, water absorption, and SHC. Trivedi and Parameshwaran^18^ used an in-situ polymerization technique to encapsulate organic ester as the core PCM within a melamine–formaldehyde shell at varying shell-to-core ratios. The resulting microcapsules exhibited a favorable latent heat and demonstrated thermal stability up to 160 °C. The MPCM suspensions also displayed low viscosity and exhibited Newtonian flow behavior, suggesting their suitability for cool TES applications.

The advent of artificial intelligence (AI) and its prominent subset, machine learning (ML), has brought about significant advancements in comprehending diverse phenomena across various disciplines^19–21^. ML algorithms empower researchers to analyze intricate datasets, extract patterns, optimize complex processes, and facilitate valuable insights and precise predictions^22–24^. Accurately predicting the TPPs of MPCMs and MPCM-based solutions is of great importance for optimizing their performance and ensuring their successful integration into practical applications. Machine learning models can assist in identifying the key factors influencing the properties of MPCMs, thereby guiding the design and synthesis process. Moreover, these models can help researchers explore various parameters and formulations, reducing the need for extensive and expensive experimental trials.

In recent years, the scientific community has observed a notable proliferation of machine learning-based techniques across diverse disciplines, encompassing MPCM-based suspensions. Ho et al.^25^ analyzed the TPPs of water-based n-eicosane MPCM solution for TES applications. The research focused on determining the TC and DV of the MPCM-based solution at various MPCM concentrations (2–10 wt%) and temperatures ranging from 25 to 50 °C. An ANN was employed to estimate the TC and DV accurately. Notably, a neural network architecture with a 2–4–4–2 structure demonstrated exceptional predictive performance, closely aligning with the experimental results. Marani et al.^26^ proposed a deep learning approach for the modeling of hydration in cementitious systems incorporating MPCMs. Their study aimed to determine the apparent activation energy associated with the hydration process. The findings of their study demonstrated the superior predictive performance of the deep neural network over the gradient-boosting ensemble method in forecasting the cumulative heat and hydration rate in cementitious systems. In another work, Marani et al.^27^ introduced a mixed ML-based procedure for concrete incorporating MPCMs. They employed a tabular generative adversarial network (TGAN) to generate synthetic mixture design data and developed robust predictive ML models. By enhancing the TGAN-GBR model with the particle swarm optimization (PSO), they optimized the mixture design of concrete and mortar with various MPCMs. Tanyildizi et al.^28^ proposed a deep-learning methodology to predict the compressive strength of cementitious composites integrated with MPCMs. They developed purposeful models using experimental datasets, including XGBoost and deep learning. The models effectively predicted compressive strength with low error and identified influential parameters for mixture design, aiding concrete development incorporating MPCM.

**Fig. 1:**
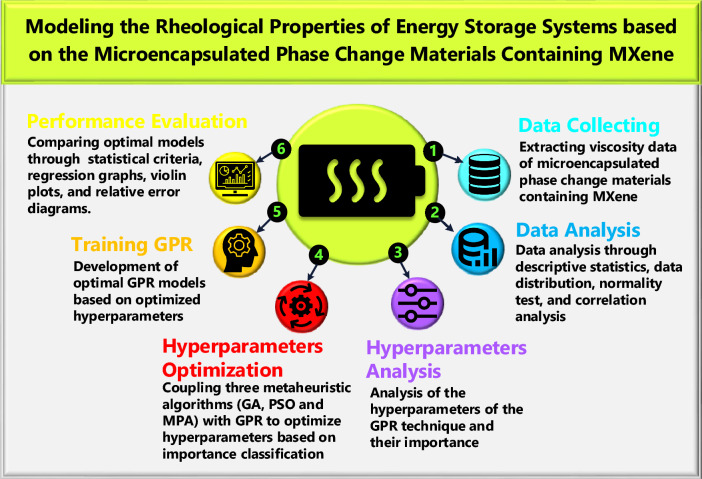
Concise overview of the modeling process employed in the present study.

Based on reviewed research, it is clear that MPCM-based suspensions hold immense potential for various applications. However, the prediction of their TPPs has been overlooked by researchers, creating a significant research gap that needs to be addressed. On the other hand, machine learning methods like Gaussian process regression (GPR) offer great power, but their effectiveness hinges on carefully chosen hyperparameters (HPs). Existing research lacks extensive sensitivity analysis on these hyperparameters. The authors of this study have directed their attention toward addressing these two concerns by focusing on the precise GPR-based prediction of dynamic viscosity in MPCM suspensions, which play a vital role in the design of energy storage systems. For this purpose, the present research introduces a comprehensive three-step procedure for identifying the crucial hyperparameters in GPR. It employs three metaheuristic algorithms (marine predators algorithm, particle swarm optimization, and genetic algorithm) for each step, offering a novel framework for optimizing hyperparameter selection in complex machine learning methods with numerous hyperparameters. The models developed in this research offer engineers a profound understanding of the dynamic viscosity of pure and nano-enhanced MPCM suspensions. The proposed novel framework offers a tool for diagnosing and optimizing HPs in ML methods involving several hyperparameters. These advancements have significant implications for enhancing efficiency across various industries, ranging from TES systems to thermal management mechanisms. Moreover, the developed models substantially decrease the expenses of experimental research and computational simulations, rendering them efficient and cost-effective solutions. Figure 1 presents a concise overview of the modeling process employed in the present study. Following the discussion of the research background and motivation in Section "Introduction", Section "Data analysis" will delve into the datasets employed in the study and the methods used for data analysis. Section "Methodology" will then review the research methodology, including the theoretical foundation of the metaheuristic algorithms and the machine learning method. Section "Models development" will focus on the model development strategy, presenting the procedure flowchart and providing specific details regarding the implementation of the modeling process. To ensure a clear evaluation of the models' performance, Section "Evaluation criteria" will describe the statistical criteria and parameters. Section "Results and discussion" will then present the overall and detailed evaluations of the developed models, including a comparative analysis to highlight subtle differences. Finally, Section "Conclusion" will conclude the paper by summarizing the key findings of the research.

## Data analysis

In this study, the experimental data of Jin et al.^29^ are used to model the dynamic viscosity of the suspension containing MPCM. To achieve optimal dispersion stability, the researchers investigated different proportions of isopropyl alcohol (IPA) to water as base fluid. They determined that a specific mass ratio (42:58) yields the best MPCM-based suspension stability. They further examined the effects of MPCM concentrations (5–15 wt%) and MXene concentrations (0.01–0.5 wt%) on improving the thermophysical properties. The suspensions underwent rigorous testing within a temperature range of 20 to 60 °C to assess their performance.

Table 1 shows the statistical specifications of the laboratory datasets. The statistical analysis of the provided data reveals valuable insights into each parameter's distribution, central tendency, variability, and shape^30^. Regarding temperature (°C), the data shows a relatively symmetrical distribution with a mean and median of 40 °C. The temperature values range from 20 °C to 60 °C, indicating a moderate spread. The standard deviation of 14.302 suggests a considerable variability in temperature measurements. The skewness of 0 indicates a lack of significant skew, while the negative kurtosis of − 1.311 implies a slightly flatter distribution compared to a normal distribution. The Kolmogorov–Smirnov statistic of 0.158 suggests a relatively small deviation from a normal distribution.

**Table 1:** Statistical characteristics of experimental data.

Item	T (°C)	MPCM MF (wt%)	MXene MF (wt%)	DV (mPa s)
Minimum	20	0	0	1.0080
Maximum	60	15	0.5	16.1990
Mean	40	8.889	0.1067	4.2101
Median	40	10	0.01	3.2449
Variance	204.55	15.78	0.0284	8.1662
Average deviation	12	2.8395	0.1304	2.1047
Standard deviation	14.302	3.973	0.1686	2.8577
Skew	0	− 0.995	1.541	2.036
Kurtosis	− 1.311	0.902	0.965	5.755
Kolmogorov–Smirnov stat	0.158	0.388	0.298	0.31

For MPCM MF (wt%), the data displays a left-skewed distribution with a mean of 8.889% and a median of 10%. The weight percentages range from 0 to 15%, indicating a moderate spread. The standard deviation of 3.973 reflects a notable variability in MPCM weight measurements. The positive kurtosis of 0.902 demonstrates a relatively peaked distribution, while the negative skewness of − 0.995 suggests a slight asymmetry towards lower values. The Kolmogorov–Smirnov statistic of 0.388 implies a moderate deviation from a normal distribution.

In the case of MXene MF (wt%), the data exhibits a right-skewed distribution with a mean of 0.1067% and a median of 0.01%. The weight percentages vary between 0% and 0.5%, indicating a relatively narrow spread. The standard deviation of 0.1686 suggests a relatively low variability in MXene weight measurements. The positive skewness of 1.541 indicates a clear asymmetry towards higher values, while the positive kurtosis of 0.965 suggests a relatively peaked distribution. The Kolmogorov–Smirnov statistic of 0.298 implies a moderate deviation from a normal distribution.

Concerning dynamic viscosity (mPa·s), the data demonstrates a right-skewed distribution with a mean of 4.2101 mPa·s and a median of 3.2449 mPa·s. The dynamic viscosity values range from 1.0080 to 16.1990 mPa·s, indicating a widespread. The standard deviation of 2.8577 reflects a moderate variability in DV measurements. The positive skewness of 2.036 suggests a clear asymmetry towards higher values, while the positive kurtosis of 5.755 demonstrates a relatively peaked distribution. The Kolmogorov–Smirnov statistic of 0.31 implies a moderate deviation from a normal distribution.

**Fig. 2:**
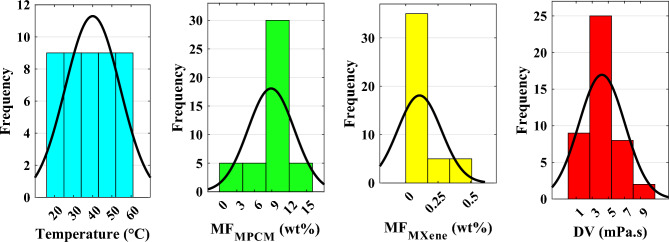
Frequency histogram of the parameters.

**Fig. 3:**
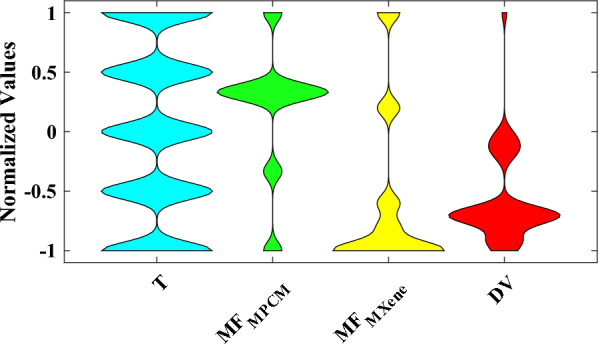
Violin graphs of the parameters.

Figure 2 shows the frequency histogram of all parameters. Visualizing the data through histograms can provide a clearer understanding of each variable's distribution patterns and frequency of measurements. The graphic representation of Fig. 2 for different parameters is entirely consistent with the findings of Table 1. In addition, utilizing violin plots can facilitate a more profound comprehension and enhanced insight into the data. As shown in Fig. 3, they combine a box plot and kernel density plot, allowing for the simultaneous display of distribution shape, central tendency, and variability. Violin plots also enable easy comparison between multiple groups or categories and can reveal multimodal or skewed distributions. This visual confirmation can complement the statistical analysis in Table 1 and help draw more robust conclusions from the data. In order to integrate all parameters into a single violin graph, data normalization is employed as follows:

1 $${x}_{norm}=\frac{x-{x}_{min}}{{x}_{max}-{x}_{min}}\times 2-1$$

The Pearson Correlation Coefficient (PCC) assesses the quantitative and qualitative relationship between dynamic viscosity and independent variables^31^. PCC is a statistical measure that assesses the linear relationship between two variables. It quantifies the strength and direction of the association, ranging from − 1 to 1. The PCC's objective is to identify and quantify the extent of linear association and facilitate data analysis. PCC is quantified according to the following relationship^31,32^:

2 $$PCC=\frac{\sum_{i=1}^{N}\left({X}_{i}-\overline{X }\right)\left({Y}_{i}-\overline{{Y }_{i}}\right)}{\sqrt{\sum_{i=1}^{N}{\left({X}_{i}-\overline{X }\right)}^{2}\sum_{i=1}^{N}{\left({Y}_{i}-\overline{{Y }_{i}}\right)}^{2}}}$$

**Fig. 4:**
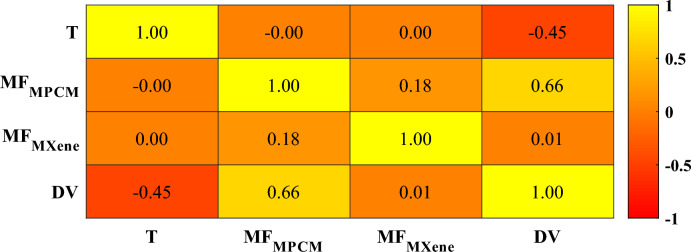
PCC between various variables.

Figure 4 shows the PCC values between different variables. According to the correlogram, dynamic viscosity is moderately negatively correlated with temperature (− 0.45), strongly positively correlated with MPCM MF (0.66), and negligibly correlated with MXene MF (0.01). This means that as temperature increases, viscosity tends to decrease. Higher MPCM MF values are associated with higher viscosity, while no significant linear relationship exists between viscosity and MXene MF. These correlations provide valuable insights into the interdependencies between these variables, aiding in understanding their behavior and potential impact on each other. While PCC values provide insights into the linear relationship between variables, it is crucial to acknowledge that there could be a significant non-linear relationship between them^33^.

## Methodology

This research uses the GPR method to model dynamic viscosity in terms of independent variables. The genetic algorithm (GA), marine predators algorithm (MPA), and particle swarm optimization (PSO) algorithm are used to optimize the HPs of the GPR model. The rest of this section introduces the theory of modeling and optimization algorithms in detail.

### Gaussian process regression

GPR is a potent technique in ML that offers a flexible and robust approach for modeling and predicting data^23^. It treats functions as random variables and assumes that any finite set of variables follows a joint Gaussian distribution (JGD). This characteristic enables GPR to capture intricate relationships and provide uncertainty estimates. The core process involves specifying a prior belief about the underlying function, typically assumed to follow a Gaussian distribution (GD)^34^. This prior belief is updated by incorporating observed data to obtain a posterior distribution, representing an improved understanding of the function given the data. This posterior distribution allows predictions to be made at new, unseen points in the dataset. What sets GPR apart is its non-parametric nature, as it does not require making assumptions about a fixed parametric form for the underlying function. This adaptability enables it to model various functions, including those with highly non-linear relationships.

Consider a set of data points, $\left\{\left({x}_{i},{y}_{i}\right);i=\text{1,2},\dots ,n\right\}$, where ${x}_{i}\in {\mathbb{R}}^{d}$ and ${y}_{i}\in {\mathbb{R}}^{d}$. It is assumed that these data points originate from an unknown distribution. When data points are utilized for training in the Gaussian process regression, a predictive model is constructed. This model aims to estimate the output vector (${y}_{new}$) corresponding to a given input vector (${x}_{new}$)^23,35^:

3 $$y= {x}^{T}\beta +\varepsilon$$

The term "$\beta$" is calculated in the training process. The term "$\varepsilon$" represents an error or noise term in the model:

4 $$\varepsilon \sim N\left(0,{\sigma }^{2}\right)$$

The estimation of error variance (${\sigma }^{2}$) is derived from the available data. In Gaussian process regression, the prediction of outputs relies on the utilization of the basis functions (BF) and covariance functions (CF) of latent variables ($f\left({x}_{i}\right),i=\text{1,2},\dots ,n$) within a Gaussian process (GP) framework. The BF, $h\left(x\right)$, serve to transform the input vector into a p-dimensional feature space, and the CF assesses the strainer properties of the corresponding outputs. The GP comprises a collection of random parameters, each selected from a JGD. Mathematically, when $\left\{f\left(x\right),x\in {\mathbb{R}}^{d}\right\}$ is regarded as a GP, the joint distribution of ${f(x}_{1})$, ${f(x}_{2})$, …, $f({x}_{n})$ is Gaussian, where ${x}_{1}$, ${x}_{2}$, …, ${x}_{n}$ represent the input variables^23,35^.

A GP is characterized by its CF, $k(x,{x}{\prime})$, and mean function (MF), $m(x)$. In other words, if we consider $\left\{f\left(x\right),x\in {\mathbb{R}}^{d}\right\}$ as a GP^23,35^:

5 $$E\left(f\left(x\right)\right)= m(x)$$

6 $$Cov\left[f\left(x\right),f\left({x}{\prime}\right)\right]= E\left[\left\{f\left(x\right)-m(x)\right\}\left\{f\left({x}{\prime}\right)-m({x}{\prime})\right\}\right]=k(x,{x}{\prime})$$

The GP model, given by $h{\left(x\right)}^{T}\beta +f(x)$, where $f(x)\sim GP\left(0,k(x,{x}{\prime})\right)$, provides a framework to model the output vector y in the following manner^23,35^:

7 $$P\left({y}_{i}\left|f\left({x}_{i}\right),{x}_{i}\right.\right) \sim N\left(y\left|H\beta +f,{\sigma }^{2}I\right.\right)$$

where

8 $$X=\left(\begin{array}{c}{x}_{1}^{T}\\ {x}_{2}^{T}\\ \vdots \\ {x}_{n}^{T}\end{array}\right), y=\left(\begin{array}{c}{y}_{1}\\ {y}_{2}\\ \vdots \\ {y}_{n}\end{array}\right), H=\left(\begin{array}{c}h\left({x}_{1}^{T}\right)\\ h\left({x}_{2}^{T}\right)\\ \vdots \\ h\left({x}_{n}^{T}\right)\end{array}\right), f=\left(\begin{array}{c}f\left({x}_{1}\right)\\ f\left({x}_{2}\right)\\ \vdots \\ f\left({x}_{n}\right)\end{array}\right)$$

The model exhibits a JGD, which can be described as follows:

9 $$P\left(f\left|X\right.\right) \sim N\left(f\left|0,K(X,X)\right.\right)$$

where,

10 $$K(X,X)=\left(\begin{array}{c}k({x}_{1},{x}_{1})\\ k({x}_{2},{x}_{1})\\ \vdots \\ k({x}_{n},{x}_{1})\end{array} \begin{array}{c}k({x}_{1},{x}_{2})\\ k({x}_{2},{x}_{2})\\ \vdots \\ k({x}_{n},{x}_{2})\end{array} \begin{array}{c}\dots \\ \dots \\ \vdots \\ \dots \end{array} \begin{array}{c}k({x}_{1},{x}_{n})\\ k({x}_{2},{x}_{n})\\ \vdots \\ k({x}_{n},{x}_{n})\end{array}\right)$$

The CF, $k(x,{x}{\prime}\left|\theta \right.)$, is characterized by a collection of kernel parameters ($\theta$).

**Fig. 5:**
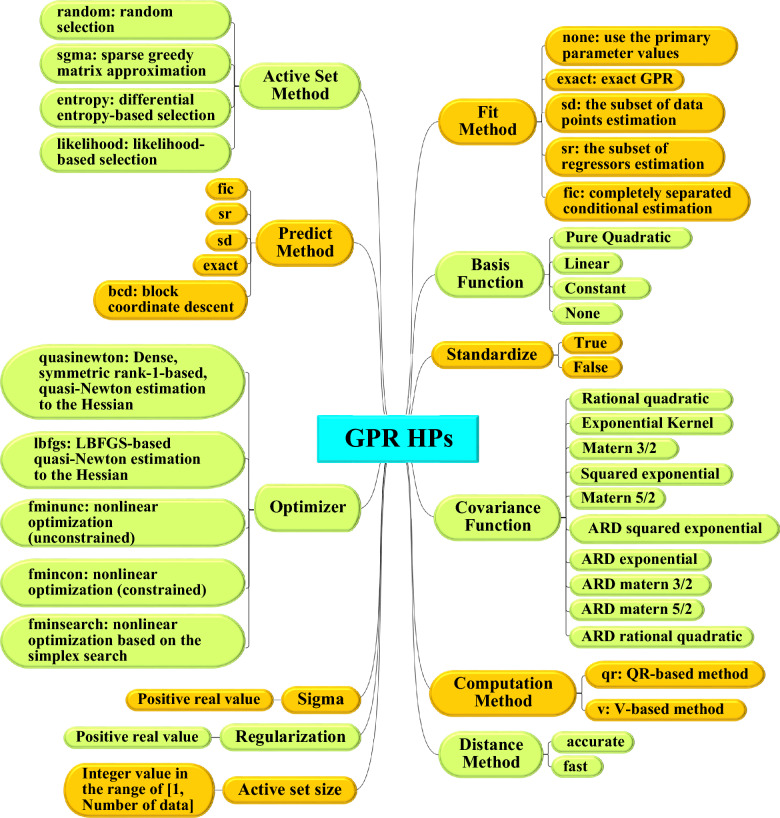
Hyperparameters of GPR method.

The GPR model provides a versatile framework for modeling and prediction tasks, offering a wide range of HPs that greatly influence the accuracy and reliability of its outputs. The selection of these parameters and configurations is crucial and should be tailored to the specific problem, taking into account the unique specifications and requirements of the task. A comprehensive overview of these HPs can be found in Fig. 5.

### Genetic algorithm

The genetic algorithm leverages a bio-inspired approach to tackle intricate optimization challenges^36^. It iteratively explores a solution landscape, mimicking the process of natural selection and evolution. This approach can be broken down into distinct phases:

1. *Population seeding* An initial population of candidate solutions (individuals) is generated within the defined parameter space. These individuals represent potential answers to the optimization problem.
2. *Fitness assessment* Each individual's efficacy is evaluated using an objective function. This function quantifies how well a particular solution aligns with the desired outcome.
3. *Selection, recombination, and mutation* A suite of operators are employed to manipulate the population and generate novel candidate solutions. Selection prioritizes more effective individuals for reproduction, recombination combines elements from selected individuals, and mutation introduces random variations to maintain population diversity.

This iterative cycle of evaluation and manipulation (steps 2 and 3) continues until a pre-defined termination criterion is reached^37^. Through these repeated steps, the GA progressively refines the population, ultimately converging on solutions with superior effectiveness as determined by the objective function. The GA's strength lies in its ability to efficiently explore a vast solution space while ensuring population diversity, thus preventing premature convergence to suboptimal solutions, a common pitfall in optimization algorithms.

In-depth analyses of the genetic algorithm, including its theoretical background and implementation details, are provided in^38–41^.

### Particle swarm optimization

The particle swarm optimization algorithm draws inspiration from the collective movement patterns observed in animal groups like flocks or fish schools^42,43^. These natural systems exhibit remarkable efficiency in navigating intricate environments, a concept that PSO utilizes to address optimization challenges. Unlike traditional methods prone to difficulties in non-linear and non-convex search spaces, PSO excels at finding solutions very close to the optimal value within such scenarios^44^. Its power lies in leveraging the "collective intelligence" of a population of virtual particles as they cooperatively search for optimal solutions^45^. The PSO algorithm operates through a repetitive process^42,45,46^:

1. *Seeding the swarm* Initially, a swarm of particles is randomly distributed within the designated search space. Each particle possesses an associated velocity.
2. *Fitness assessment* The effectiveness of each particle is evaluated based on the problem's objective function.
3. *Velocity and position updates* Particles update their velocities and positions considering two crucial factors:
4. *Individual memory* Each particle retains a memory of its own most effective position encountered so far (representing its own learning).
5. *Swarm knowledge* Particles are also influenced by the most effective position discovered by any particle within the swarm (representing a form of social learning). This information guides their movement towards promising regions of the search space.
6. *Iterative refinement* The process continues iteratively as particles update their positions and velocities, gradually converging towards optimal solutions. A pre-defined stopping criterion, such as reaching a maximum number of iterations or achieving a desired level of effectiveness, determines the algorithm's termination.
7. *Optimal candidate identification* The particle with the highest fitness value throughout the optimization process represents the PSO algorithm's final output, signifying the solution closest to the optimum.

PSO's strength lies in its ability to maintain a balance between exploration (searching new areas) and exploitation (focusing on promising regions) through the combined influence of individual and collective memory. This dynamic approach allows PSO to effectively navigate complex search spaces and identify solutions very close to the optimal value.

References^43,46–48^ offer detailed discussions on the particle swarm optimization, covering both its theoretical underpinnings and implementation intricacies.

### Marine predators algorithm

The MPA is a nature-inspired optimization algorithm that mimics the hunting behavior of marine predators, such as sharks, in an aquatic ecosystem. Faramarzi et al.^49^ introduced the MPA in 2020, which has since gained recognition for its remarkable capability to efficiently explore and exploit search spaces. MPA has demonstrated its effectiveness, particularly in tackling intricate optimization problems^50^.

In order to comprehend the intricacies of this algorithm, it is crucial to delve into its foundation, which lies in the hunting behavior of marine predators. The Lévy strategy is commonly observed among marine predators, such as sharks, especially in prey-scarce environments. However, when these predators encounter areas abundant with prey, they predominantly adopt a Brownian motion pattern characterized by random movement. This switch in foraging behavior highlights their adaptability to different ecological conditions and the ability to optimize their search strategies based on prey availability^51^. The type of movement adopted by each participant, whether a predator or prey, plays a significant role in determining the most effective approach for encounters. Additionally, the relative velocities between the predator and prey further shape the encounter rate policy^52^. The following summarization encapsulates the key governing policies that drive optimal foraging strategies, interactions, and memory formation in marine predators^49^:

- Marine predators employ the Lévy strategy when navigating environments with low prey concentrations, optimizing their search patterns for efficient foraging. However, in areas abundant with prey, they predominantly switch to the Brownian movement.
- Marine predators exhibit consistent proportions of Lévy and Brownian movement patterns throughout their lifetime as they traverse diverse habitats.
- Marine predators exhibit changes in their behavior in response to environmental factors, including both natural phenomena such as eddy formation and anthropogenic influences like fish aggregating devices (FADs).
- The optimal predator strategy in scenarios characterized by a low-velocity ratio (v = 0.1) is the Lévy movement. This holds irrespective of whether the prey exhibits Brownian or Lévy movement patterns.
- In the case of a unit velocity ratio (v = 1), the optimal strategy for the predator is Brownian movement when the prey exhibits Lévy motion.
- In situations characterized by a high-velocity ratio (v = 10), the optimal strategy for the predator is to remain stationary, regardless of whether the prey exhibits Brownian or Lévy movement patterns.
- Marine predators leverage their advanced memory capabilities to their advantage, utilizing recollection of their conspecifics and successful foraging opportunities' locations.

Drawing upon these key observations, the optimization process of MPA can be delineated into three distinct phases. The first phase (exploration phase) pertains to high-velocity ratios, where prey outpaces the predator. The second phase (transition phase) encompasses unit velocity ratios, wherein predator and prey move at nearly equivalent speeds. Finally, the third (exploitative phase) addresses low-velocity ratios, where the predator exhibits greater velocity than the prey. In addition to the three distinct phases outlined for optimization, the inclusion of natural and anthropogenic environmental factors is crucial in the development of MPA. All these factors are mathematically modeled to incorporate their effects within the MPA framework.

An extensive investigation into the marine predators algorithm is provided in references^49,50,53,54^. These explorations delve into the theoretical principles upon which MPA is based and the intricacies involved in its practical implementation.

## Models development

By systematically exploring and evaluating different HP settings, we can gain valuable insights into the impact of these parameters on the ML algorithms' performance. Indeed, finding the optimal combination of HPs that influence machine learning algorithms' training and structural aspects can be challenging and complex. Without a principled method or systematic approach, achieving this optimal combination may prove difficult, if not impossible. The interplay between different hyperparameters and their effects on the algorithm's performance can be intricate and non-intuitive.

In situations where finding the optimal set of HPs for ML algorithms is challenging, utilizing metaheuristic algorithms (GA, PSO, and MPA) can offer an intelligent solution. As depicted in Fig. 5, the present study focuses on examining the impact of 12 distinct HPs on the performance of the GPR method. These hyperparameters encompass a range of factors, including the kernel function, optimizer, predict method, active set method, active set size, distance method, computation method, regularization, standardization, sigma, basis function, and fit method. The effects of these HPs on GPR model performance can vary depending on the nature of the problem being addressed and the characteristics of the available data.

This paper undertakes an analysis to comprehensively evaluate the impact of each HP on the performance of the data-driven model. The purpose of this analysis is to gain insight into hyperparameters. The initial step focuses on identifying the most influential HPs by assessing their individual effects on the model's performance. These constitute the first group of HPs. In the second step, the less impactful HPs are added to the first group to refine the model's performance further, referred to as the second group of HPs. Considering their combined effects, this step allows for a more comprehensive exploration of the HPs space. Finally, in the last step, all 12 HPs are considered simultaneously, called the third group. This integration enables the model to leverage the collective impact of all HPs. This research's optimization and modeling processes are conducted using MATLAB software (R2021b).

This research uses the training dataset, which constitutes 80% of the entire database, to train the machine learning algorithms. During the training phase, the model learns patterns and relationships within the data to make accurate predictions. After the training phase, the remaining 20% of the dataset, known as the testing dataset, is employed to evaluate the effectiveness of the trained models. This independent dataset allows researchers to evaluate how well the models generalize to unseen data and estimate their performance in real-world scenarios. In addition, employing specific datasets for both training and testing purposes is crucial to ensure a fair and consistent comparison between different models throughout the modeling process. Also, a subset of training data is separated as validation data to reduce the risk of model overtraining. The leave-one-out cross-validation is utilized for this purpose.

A fixed number of function evaluations (NFEs) is used to ensure a fair comparison between optimization algorithms. This approach helps to standardize the evaluation process and ensures that each algorithm is given an equal opportunity to optimize and converge within the same computational budget. In the HP optimization process, the study utilizes 100, 200, and 300 function evaluations in the first, second, and third steps to guide the exploration and refinement of HP settings. Due to the random nature of optimization algorithms and the substantial influence of the initial population's quality on the final solution, the optimization process is repeated five times for each case in this study. The best solution obtained across these five runs is then reported as the final solution.

**Fig. 6:**
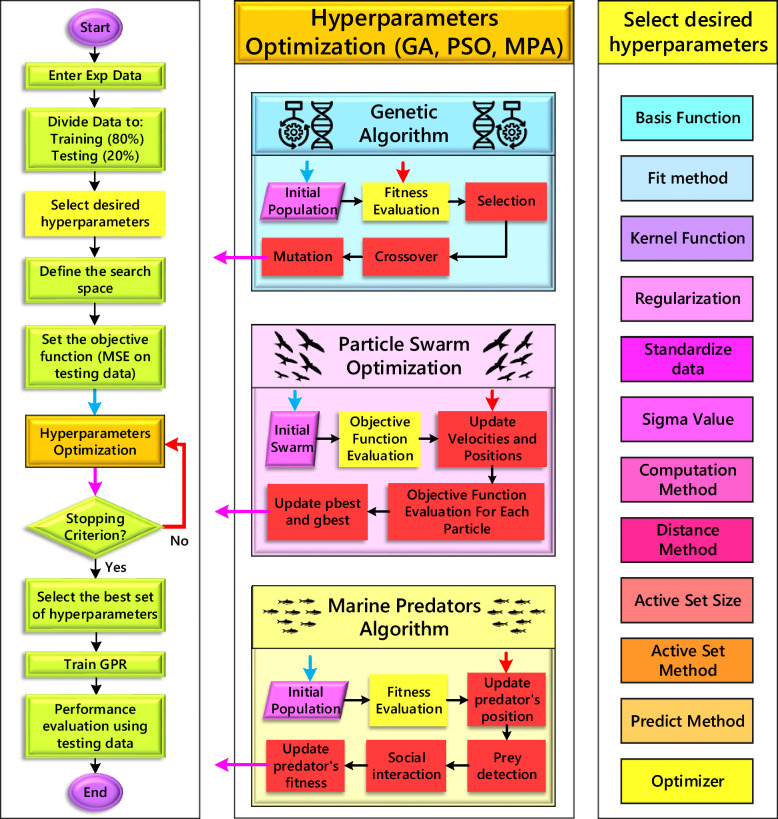
Flowchart of the present study.

The selection of metaheuristic optimization algorithms (MOAs) was guided by two key considerations. Firstly, the GA and PSO were chosen due to their well-documented effectiveness in optimizing hyperparameters for machine learning models, especially those involving mixed-integer values. Secondly, we included the MPA as a novel promising, yet under-evaluated MOA. This inclusion aimed to assess its potential for hyperparameter optimization and contribute to its broader exploration within the field. This selection strategy facilitated a two-fold benefit. It allowed for benchmarking established MOAs like GA and PSO against a novel algorithm (MPA). Additionally, it ensured a balance between exploration of diverse solution spaces and efficient refinement through the well-established search capabilities of GA and PSO. Parameter settings for the GA, PSO, and MPA algorithms can be found in Table 2. Figure 6 shows the flowchart of the present study.

**Table 2:** Parameters settings for the GA, PSO, and MPA algorithms.

GA				PSO				MPA			
Parameters	4 HPs	7 HPs	12 HPs	Parameters	4 HPs	7 HPs	12 HPs	Parameters	4 HPs	7 HPs	12 HPs
Population size	10	20	30	Swarm size	10	20	30	Agent number	5	10	15
Generations	10			Iterations	10			Iterations	10		
Crossover fraction	0.8			Minimum adaptive neighborhood size	0.25			FAD index	0.2		
Mutation function	Power			Inertia range	[0.1, 1.1]			P value	0.5		
Crossover function	Laplace			Creation function	Pswcreationuniform			Function Tolerance	1E−8		
Selection function	Tournament			Function tolerance	1E−8						
Function tolerance	1E−8

Furthermore, the fitness function for the metaheuristic algorithms is defined by the mean squared error (MSE) on the testing data set. This choice ensures that the algorithms prioritize models with strong generalization capabilities, emphasizing performance on previously unseen data, which is crucial for developing reliable regression models.

## Evaluation criteria

To evaluate the efficacy of GPR models, five statistical metrics are employed. These metrics consist of Willmott's Index of Agreement (I_A_), Coefficient of Determination (R^2^), Correlation Coefficient (R), Mean Absolute Percentage Error (MAPE), and Mean Squared Error (MSE)^55,56^:

11 $$MSE=\frac{1}{n}\sum_{i=1}^{n}{\left({Y}_{i,Exp}-{Y}_{i,Pred}\right)}^{2}$$

12 $$MAPE\left(\%\right)=\frac{1}{n}\sum_{i=1}^{n}\left|\frac{{Y}_{i,Pred}-{Y}_{i,Exp}}{{Y}_{i,Exp}}\right|\times 100$$

13 $$R=\frac{\sum_{i=1}^{n}\left({Y}_{i,Exp}-\overline{{Y }_{i,Exp}}\right)\left({Y}_{i,Pred}-\overline{{Y }_{i,Pred}}\right)}{\sqrt{\sum_{i=1}^{n}{\left({Y}_{i,Exp}-\overline{{Y }_{i,Exp}}\right)}^{2}\sum_{i=1}^{n}{\left({Y}_{i,Pred}-\overline{{Y }_{i,Pred}}\right)}^{2}}}$$

14 $${R}^{2}=1-\sum_{i=1}^{n}\frac{{\left({Y}_{i,Pred}-{Y}_{i,Exp}\right)}^{2}}{{Y}_{i,Exp}^{2}}$$

15 $${I}_{A}=1-\frac{\sum_{i=1}^{n}{\left({Y}_{i,Exp}-{Y}_{i,Pred}\right)}^{2}}{\sum_{i=1}^{n}{\left(\left|{Y}_{i,Pred}-\overline{{Y }_{i,Exp}}\right|+\left|{Y}_{i,Exp}-\overline{{Y }_{i,Exp}}\right|\right)}^{2}}$$

In the formulas, the symbol "n" represents the total number of data points. The notation "${Y}_{i,Pred}$" denotes the predicted values, while "${Y}_{i,Exp}$" represents the corresponding experimental values.

The MSE and MAPE metrics provide a measure of the error magnitude associated with the predictions. A lower value of MAPE and MSE indicates a higher level of accuracy and validity in the model's predictions. On the other hand, R, R^2^, and I_A_ criteria produce a value between 0 and 1, with a value closer to 1 suggesting a strong agreement and validity in the predictions.

Alongside the aforementioned criteria, two additional metrics, Absolute Relative Deviation (ARD) and Relative Error (RE), are employed to graphical analyze the accuracy of outputs:

16 $${ARD}_{i}\left(\%\right)=\left|\frac{{Y}_{i,Exp}-{Y}_{i,Pred}}{{Y}_{i,Exp}}\right|\times 100$$

17 $${RE}_{i}\left(\%\right)=\left(\frac{{Y}_{i,Exp}-{Y}_{i,Pred}}{{Y}_{i,Exp}}\right)\times 100$$

## Results and discussion

### Influence of various HPs

This section aims to analyze the influence of each hyperparameter. The impact of different HPs on the model's performance will be discussed in detail. Subsequently, the HPs will be categorized into three distinct groups based on their effectiveness. Each group will be optimized separately, focusing on fine-tuning the HPs within that specific category. The HPs are classified into three groups based on their susceptibility and effectiveness. The first group comprises the most susceptible HPs, which are highly sensitive and can significantly impact the model's performance. The second category consists of HPs, the first group plus those identified as effective though insignificant in improving the model's performance. Finally, the third category encompasses all the HPs.

The effectiveness of each hyperparameter is assessed by observing its impact on the model's performance. This evaluation is done by measuring the change in the model's performance (R^2^ on the testing data) when different options or values of the hyperparameter are applied to the default model. The default model refers to a state where no specific optimization or principled selection has been applied to the HPs. In this context, the HPs are initialized with default values provided by MATLAB and listed in Table 3. The performance of the default model is evaluated and presented in Table 4. It is evident from the results that the default model, with a MAPE of 28% and an R^2^ of approximately 0.86, exhibits poor performance when applied to the testing data. These values indicate that the default HP values do not yield satisfactory outputs for the given problem. This outcome underscores the necessity to optimize the HPs for the GPR models.

**Table 3:** Default values of HPs.

Hyperparameters	Default value
Optimizer	quasinewton
Predict Method	exact
Active Set Method	random
Active Set Size	36
Distance Method	fast
Computation Method	qr
Fit Method	exact
Standardize	false (0)
Kernel Function	squared exponential
Basis Function	constant
Sigma (std(targets)/sqrt(2))	2.02067056
Regularization (1E−2 × std(targets))	0.028576597

**Table 4:** Performance of default model on testing and training datasets.

Data	MSE	MAPE (%)	R	R^2^	I_A_
Testing	8.69753E− 01	28.083	0.927839	0.860885	0.960571
Training	3.87282E−02	3.385	0.997705	0.995416	0.998842

**Fig. 7:**
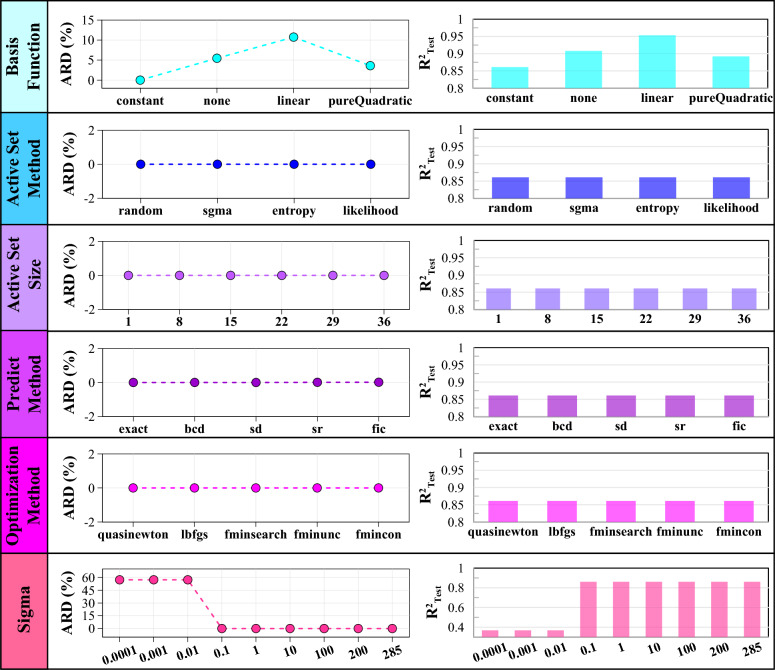
The effect of different values of basis function, active set method, active set size, predict method, optimizer, and sigma on default model performance.

**Fig. 8:**
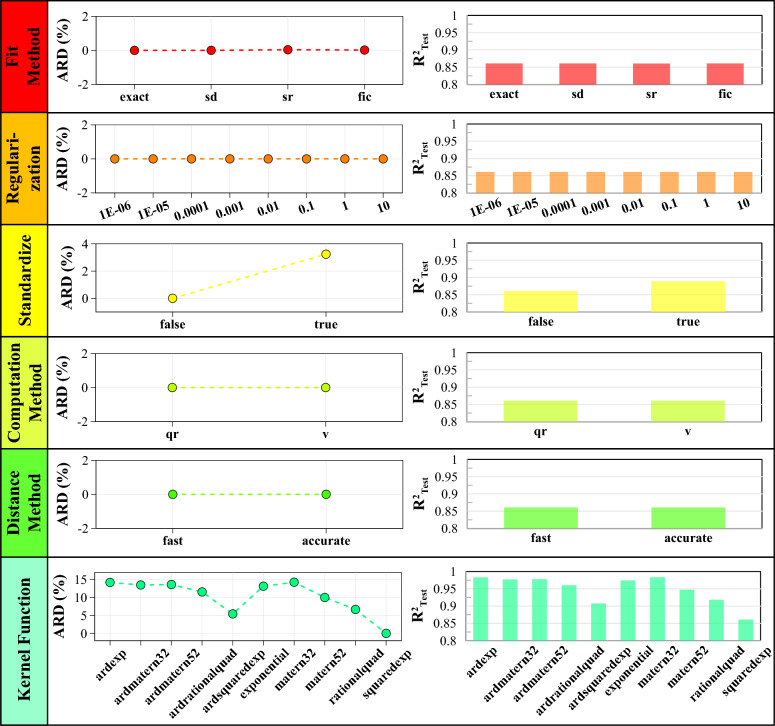
The effect of various values of fit method, regularization, standardize, computation method, distance method, and kernel function on default model performance.

Figures 7 and 8 visually represent how changes in hyperparameter values impact the model outputs' performance. By analyzing the relative deviation from the R^2^ of the default model, one can observe the extent to which each hyperparameter affects the model's accuracy. Additionally, the R^2^ of the models under different hyperparameter values provides insights into the choices for achieving improved performance.

In order to determine the HPs of the first group, a selection is made based on their effectiveness. In this case, four of the most effective HPs are chosen. Based on the analysis of the figures, it is observed that changing the covariance (kernel) function from the default value (squared exponential) significantly impacts the testing data's accuracy. It specifies how the GP model assigns uncertainty to predictions based on the proximity of input points in the feature space. The default model R^2^ value increases from 0.86 to a minimum of 0.908 (ARD squared exponential) and a maximum of 0.984 (Matérn32). The absolute relative deviation from changing the kernel function ranges from 5.43% to 14.28%. Additionally, changing the basis function can enhance the model's performance by a range of 3.6% to 10.74%. The change of sigma, which represents the initial value for the noise standard deviation of the GPR model, can have a notable effect on the model's performance. Figure 7 demonstrates that the choice of sigma values greater than 0.1 has a negligible effect on the model output. The model's outputs remain relatively stable, and the R^2^ value remains close to the initial value of 0.86. However, when sigma values are less than 0.01, the model's efficiency is drastically affected. The R^2^ value decreases significantly from 0.86 to 0.368. The parameter "standardize" plays a crucial role in evaluating the impact of data standardization on the model. When set to "true", it indicates that the data will undergo a standardization process. This process involves centering and scaling each dataset column based on the mean and standard deviation. In the default mode, standardization is not performed (standardize = "false"). However, with standardization for the present data, there is an improvement in the accuracy of the output model (3.24%).

The second group of parameters includes the four previously mentioned effective parameters and additional parameters that, while insignificant, are still considered. These HPs are the fit method, predict method, and optimizer. The optimizer encompasses a set of optimization techniques utilized to compute model parameters. The predict method outlines the procedure for generating predictions based on the GP samples. The estimation of parameters in the GPR model is performed using the fit method. Based on the Figs. 7 and 8, changing the values of the fit method, predict method, and optimizer has a relatively small impact on the model's accuracy (less than 1%).

Furthermore, changing the values of HPs, such as active set method, active set size, distance method, computation method, and regularization, does not affect the precision of the base (default) model. In GPR, the active set method assigns observations to vectors during the model training. The active set size determines the number of observations included in the active set during the training process. The active set size is an integer value from 1 to the number of data points. The distance method calculates the inter-point lengths between data points when evaluating kernel functions in GP models. The computation method calculates the log-likelihood and gradient in GP models. Finally, regularization is a fundamental concept within GP models that plays a crucial role in mitigating overfitting and enhancing the model's generalization capability. It involves adding a regularization term to the log-likelihood function, encouraging smoothness and simplicity in the estimated functions. It should be noted that the effect of these HPs should not be underestimated because the different HPs can interact with each other, and changing one HP may impact the optimal values of other HPs. Hence, the third group combines the HPs of the first and second groups, along with the five HPs mentioned. Considering all these parameters makes the intricate interplay between them in the modeling process evident.

### Evaluation of various categories of HPs

Table 5 shows the results of optimized models by metaheuristic algorithms for the first group of HPs, which include four critical HPs. Based on the information provided in Table 5, the three algorithms used to optimize the HPs have achieved very similar performance results. The reported values of 0.998303, 0.996608, and 0.999135 for the R, R^2^, and I_A_ indicate the reasonable performance of all three optimization algorithms. In contrast, a subtle discrepancy becomes apparent among the models when considering the aspect of error. Specifically, the MPA-GPR model exhibits a lower MAPE when evaluated on the testing dataset, albeit with a marginal variance compared to the other two models. The subtle divergence observed in the models' responses can be attributed to the sigma value optimized through distinct algorithms. As evidenced by Table 6, the MPA algorithm yielded a lower estimated sigma value despite employing identical configurations of the basis function and kernel function and standardized as the other algorithms.

**Table 5:** Performance of optimized models (4 HPs) on testing and training datasets. The superior optimization technique is in bold.

Data	Optimization technique	MSE	MAPE (%)	R	R^2^	I_A_
Testing	GA	2.05989E−02	5.123125	0.998303	0.996608	0.999135
	PSO	2.05990E−02	5.123127	0.998303	0.996608	0.999135
	**MPA**	**2.05989E−02**	**5.123120**	**0.998303**	**0.996608**	**0.999135**
Training	GA	1.14049E−04	0.105840	0.999993	0.999986	0.999997
	PSO	1.14049E−04	0.105841	0.999993	0.999986	0.999997
	MPA	1.14051E−04	0.105842	0.999993	0.999986	0.999997

**Table 6:** Values of optimized HPs for first group (4 HPs).

Basis function	Kernel function	Standardize	Sigma (GA)	Sigma (MPA)	Sigma (PSO)
None	ardmatern32	true	241.1775404	60.60151263	111.8192712

**Fig. 9:**
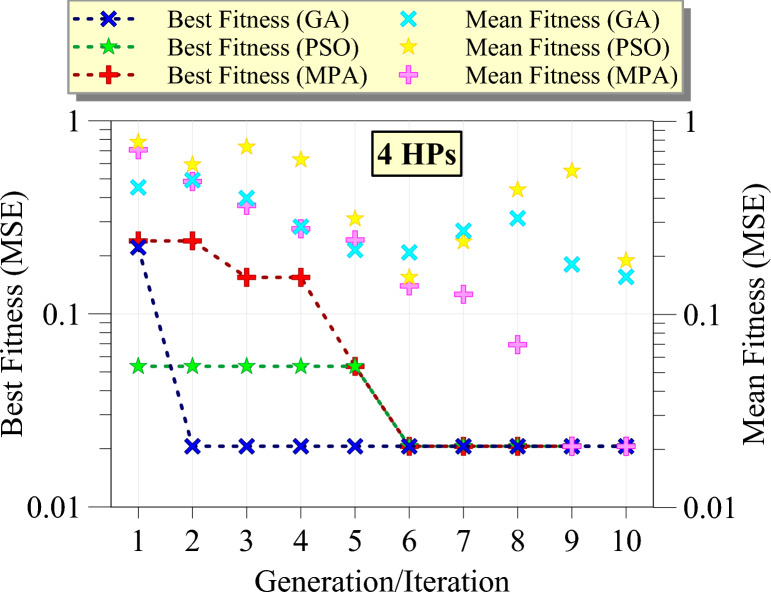
The best fitness and mean fitness values as the generation/iteration progress for different metaheuristic algorithms for the first group (4 HPs).

Figure 9 illustrates the optimization process of GPR models using three different metaheuristic algorithms for the first group of hyperparameters. The x-axis represents the number of generations or iterations, while the y-axis shows the fitness values measured by MSE on a logarithmic scale. Based on the figure, the best fitness values for GA show a significant improvement from the first generation, rapidly decreasing and stabilizing around the second generation, indicating that GA efficiently finds a near-optimal solution early in the optimization process. In contrast, the best fitness values for PSO demonstrate a favorable start, achieving a stable optimum value after the sixth iteration, reflecting a slower convergence compared to GA but reaching a comparable final fitness value. The best fitness values for MPA show a gradual improvement, taking up to the sixth iteration to converge to a stable value, indicating a slower convergence speed than both GA and PSO. The mean fitness values for GA decrease gradually across generations, showing a trend similar to the best fitness values but with higher MSE initially, indicating that GA maintains a balance between exploration and exploitation. This trend is also observed with a steeper slope for the PSO algorithm. The sudden increase in mean fitness in iterations 7–9 indicates the depth of exploration in the space of hyperparameters. For MPA, the mean fitness values show a steady decline, aligning closely with the best fitness values, suggesting that the population of solutions in MPA improves uniformly across iterations. Overall, GA is the most efficient algorithm in terms of rapid convergence and achieving low MSE for the first group of hyperparameters, with PSO also performing well but with a slower convergence rate, and MPA eventually achieving comparable performance despite its slower convergence.

A more comprehensive understanding of the performance of optimization algorithms can be attained by considering the HPs within the second group. This group encompasses seven specific HPs that undergo optimization. The outcomes of the optimal models for the testing and training data are presented in Table 7. As per the table, the models optimized with seven hyperparameters using various algorithms resemble those observed in the first group in terms of their accuracy. Nevertheless, the PSO-based model has exhibited superior performance compared to the other two algorithms in terms of key evaluation metrics such as MSE, R, R^2^, and I_A_. However, it is noteworthy that the PSO-GPR model demonstrates a weaker performance in terms of MAPE in comparison to the model based on the GA. According to the findings presented in Table 8, it is obvious that all three models exhibit similarity in their basic parameters, including basis function, kernel function, and standardization. However, distinctions arise in some HPs, including fit method, optimizer, and sigma, which account for the subtle differences observed in their performance.

**Table 7:** Performance of optimized models (7 HPs) on testing and training datasets. The superior optimization technique is in bold.

Data	Optimization technique	MSE	MAPE (%)	R	R^2^	I_A_
Testing	GA	2.03175E−02	5.105660	0.998329	0.996661	0.999147
	**PSO**	**2.01947E−02**	**5.112925**	**0.998340**	**0.996682**	**0.999153**
	MPA	2.05855E−02	5.130188	0.998303	0.996609	0.999136
Training	GA	1.16693E−04	0.108089	0.999993	0.999986	0.999997
	PSO	1.11205E−04	0.103392	0.999993	0.999987	0.999997
	MPA	1.10446E−04	0.102788	0.999993	0.999987	0.999997

**Table 8:** Values of optimized HPs for second group (7 HPs).

Optimization technique	Basis function	Kernel function	Standardize	Fit method	Predict method	Optimizer	Sigma
GA	None	ardmatern32	false	sr	sd	quasinewton	66.98239
PSO	None	ardmatern32	false	sr	sd	fminsearch	9.536867
MPA	None	ardmatern32	false	sd	sd	fminsearch	4.182643

**Fig. 10:**
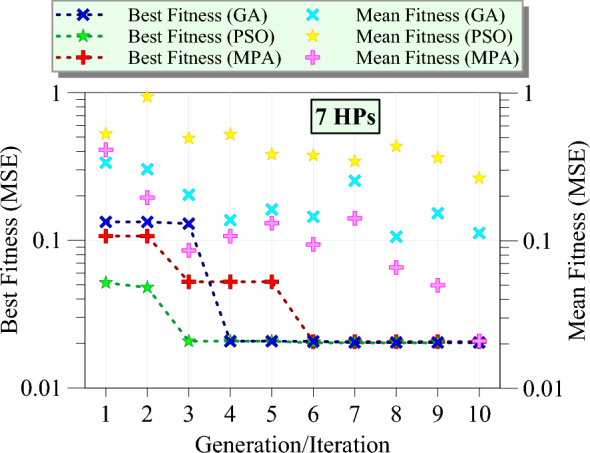
The best fitness and mean fitness values as the generation/iteration progress for different metaheuristic algorithms for the second group (7 HPs).

Figure 10 presents the HP optimization process of GPR models using GA, PSO, and MPA for the second group of HPs. The best fitness values for GA show a sharp decline from the third to the fourth generation, reaching a stable minimum value, and indicating a rapid convergence to an optimal solution. The best fitness values for PSO exhibit a faster improvement, stabilizing at a lower MSE after the second iteration, suggesting a steady convergence with consistent performance across iterations. The best fitness values for MPA demonstrate a slower initial improvement, similar to the first group, but achieve a stable and comparable minimum MSE by the sixth iteration. The mean fitness values for PSO decrease progressively, mirroring the trend of the best fitness values but showing a wider range initially, indicating PSO’s effective balance between exploration and exploitation. For MPA, the mean fitness values steadily decline, closely following the best fitness values, reflecting a uniform improvement in the solution population over iterations. GA’s mean fitness values decrease slowly similar to PSO, showing higher MSE at the seventh iteration but eventually converging to a stable value near 0.1, indicating a slower yet consistent convergence. Overall, this figure indicates that PSO remains the most efficient algorithm in terms of fast convergence and achieving low MSE, with GA and MPA also performing well but with different convergence rates.

Including all HPs in the optimization process allows for exploring their interactions and determining their mutual influence on the accuracy of the models. By considering the collective impact of these HPs, a comprehensive analysis can be conducted to unravel the intricate relationships and dependencies between them. The outcomes of the optimized models, incorporating 12 distinct HPs, are presented in Table 9. As per the table, the genetic algorithm has demonstrated an ability to identify a combination of HPs that significantly deviates from those observed in the other two models. The GA-GPR model demonstrates a remarkable level of compliance between its outputs and the respective targets, as indicated by the high values of R (0.999224) and R^2^ (0.998449) obtained on the testing data. Furthermore, the GA-GPR model exhibits a lower error rate, with a MAPE of 4.024%, which shows 1% less error than the PSO-GPR and MPA-GPR models. The list of optimized HPs for the GA-, PSO-, and MPA-based models is presented in Table 10. Based on the table, it is obvious that all three optimization algorithms exhibit agreement with each other in only four parameters: basis function, kernel function, fit method, and computation method. This finding highlights the limited consensus among the algorithms regarding the optimal values for most HPs. Furthermore, the optimization of hyperparameters using GA, PSO, and MPA significantly altered the settings compared to the default values, enhancing the model's accuracy. Based on Table 3, the default configuration featured a 'quasinewton' optimizer, 'exact' predict and fit methods, 'random' active set method, and a 'squared exponential' kernel function with a basis function set to 'constant'. The optimized hyperparameters varied notably across the three meta-heuristic algorithms. The basis function was uniformly changed to 'none,' and the kernel function shifted to 'ardmatern32' for all algorithms, indicating a preference for a more flexible and sophisticated kernel. For standardization, GA and PSO retained 'false,' while MPA adjusted it to 'true,' potentially improving normalization. The optimizers varied with GA using 'fminsearch,' PSO 'lbfgs,' and MPA 'fmincon,' showing diverse approaches in local and global optimization techniques. The prediction method for GA and PSO switched to 'sd,' while MPA maintained 'exact.' The active set size was reduced variably (31 for GA, 34 for PSO, and significantly to 4 for MPA), indicating different strategies in model complexity and computation. The active set method for PSO changed to 'entropy,' enhancing sample selection diversity. Both PSO and MPA shifted the distance method to 'accurate' from 'fast,' prioritizing precision over speed. The sigma values and regularization parameters also varied greatly, with GA and MPA increasing sigma values substantially (61.96242 and 266.4889, respectively), while PSO dramatically reduced it to 0.0001, and regularization increased for GA and PSO but slightly decreased for MPA. These tailored hyperparameter settings by each meta-heuristic algorithm resulted in a better-fitting and more accurate GPR model, demonstrating the efficacy of customized optimization in machine learning.

**Table 9:** Performance of optimized models (12 HPs) on testing and training datasets. The superior optimization technique is in bold.

Data	Optimization technique	MSE	MAPE (%)	R	R^2^	I_A_
Testing	**GA**	**1.49996E−02**	**4.024234**	**0.999224**	**0.998449**	**0.999355**
	PSO	2.03798E−02	5.104828	0.998323	0.996649	0.999145
	MPA	2.05858E−02	5.130193	0.998303	0.996609	0.999136
Training	GA	1.19456E−02	0.309905	0.999522	0.999044	0.999653
	PSO	2.00416E−04	0.112818	0.999989	0.999978	0.999994
	MPA	1.10446E−04	0.102788	0.999993	0.999987	0.999997

**Table 10:** Values of optimized HPs for third group (12 HPs).

Hyperparameters	GA	PSO	MPA
Basis function	None	None	None
Kernel function	ardmatern32	ardmatern32	ardmatern32
Standardize	false	false	true
Fit method	exact	exact	exact
Optimizer	fminsearch	lbfgs	fmincon
Predict method	sd	sd	exact
Active set size	31	34	4
Active set method	random	entropy	random
Distance method	fast	accurate	accurate
Computation method	qr	qr	qr
Sigma	61.96242	0.0001	266.4889
Regularization	2.045167	8.488548	0.020363

**Fig. 11:**
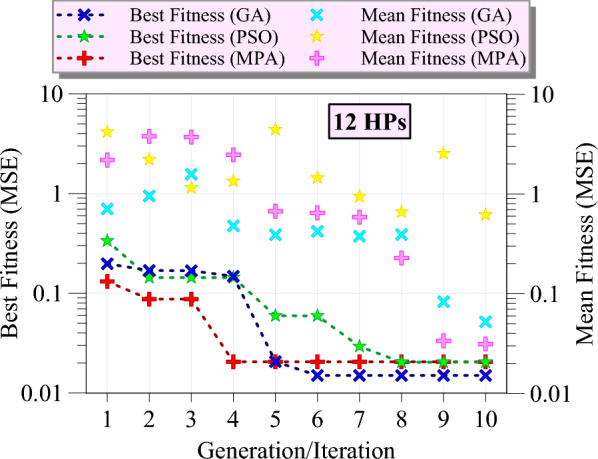
The best fitness and mean fitness values as the generation/iteration progress for different metaheuristic algorithms for the third group (12 HPs).

Figure 11 demonstrates the optimization process of GPR models for the third group of HPs. According to the figure, the best fitness values for MPA show a sharp initial decline, stabilizing at a low MSE by the fourth iteration, demonstrating MPA's rapid convergence capability. PSO exhibits a more gradual reduction in MSE, achieving stability around the eighth iteration, reflecting a steady improvement and consistent performance. GA, on the other hand, shows a slower initial improvement but ultimately reaches a stable lower MSE by the sixth generation, indicating an effective convergence. The mean fitness values for GA follow a decreasing trend, though with a broader range initially, suggesting effective exploration and subsequent exploitation of the search space. MPA’s mean fitness values decline steadily, closely aligning with the best fitness values, indicating a uniform enhancement in the population's overall performance. PSO’s mean fitness values decrease more slowly, along with the sudden increases in the average fitness value, which is caused by the comprehensive search in the hyperparameter space. Generally, GA maintains its efficiency in gradual convergence and achieves the lowest MSE. PSO shows reliable performance with steady improvement over iterations, while MPA, despite a satisfactory start, ultimately achieves the worst optimization results.

### Comparison of superior models

**Fig. 12:**
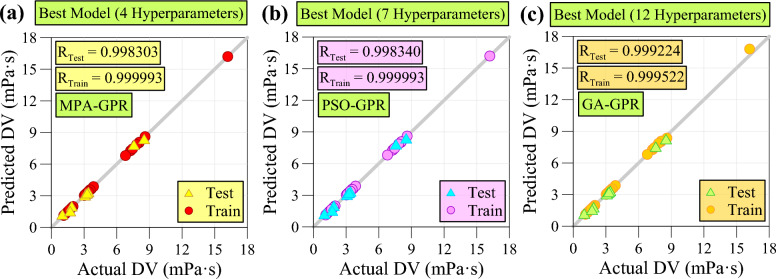
Regression diagrams for superior model of each hyperparameter groups.

The regression diagrams presented in Fig. 12 illustrate the performance of the superior model across different categories of hyperparameters. Based on Fig. 12a,b, it is apparent that both the MPA-GPR and PSO-GPR models exhibit remarkably similar performance in both the testing and training phases. Upon closer examination of the regression diagrams for the MPA-GPR and PSO-GPR models, it is evident that the training data points exhibit a strong alignment with the Y = X line, indicating a high level of compliance. However, a noticeable deviation from the Y = X line is observed for some test data points, suggesting a certain level of variability in the model's predictive accuracy for unseen data. In contrast, the model based on the genetic algorithm, which optimizes all 12 parameters, demonstrates exceptional performance in the test phase. This model exhibits a distinct advantage over the MPA-GPR and PSO-GPR models, as evidenced by its superior ability to accurately predict outcomes for unseen data. This finding highlights the effectiveness of the genetic algorithm in identifying an optimal combination of hyperparameters that enhances the model's generalization capabilities. According to Fig. 12c, the model based on the GA does exhibit a slightly weaker performance in the training phase compared to the MPA-GPR and PSO-GPR models. Some training data points show a slight deviation from the Y = X line, indicating a minor discrepancy between the predicted and actual values.

**Fig. 13:**
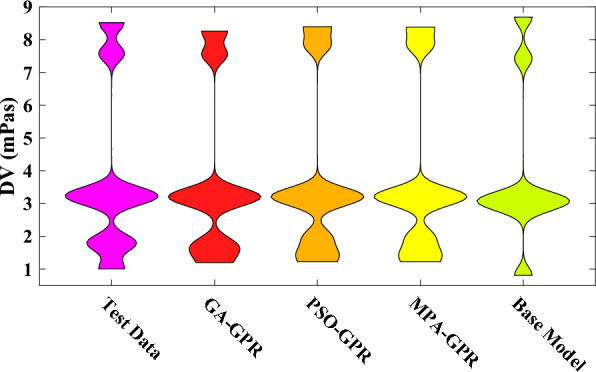
Comparison of the violin plots of the outputs of default and optimized models.

In Fig. 13, a violin plot compares the outputs of the default model, the optimized models, and the actual data in the testing phase. The shape of the density function in the plot provides insights into the similarity between the model outputs and the actual data. Notably, the outputs of the GA-GPR model exhibit the highest similarity with the actual data, as evidenced by the shape of the density function. Conversely, the default model displays the largest discrepancy with the experimental data, indicating its limited accuracy in capturing the underlying patterns. The largest differences between the predicted and actual data are observed at low and high dynamic viscosity values. The optimal models' predictions in these regions are accompanied by a more significant error, suggesting that further improvements are needed to enhance their performance in these specific ranges.

**Fig. 14:**
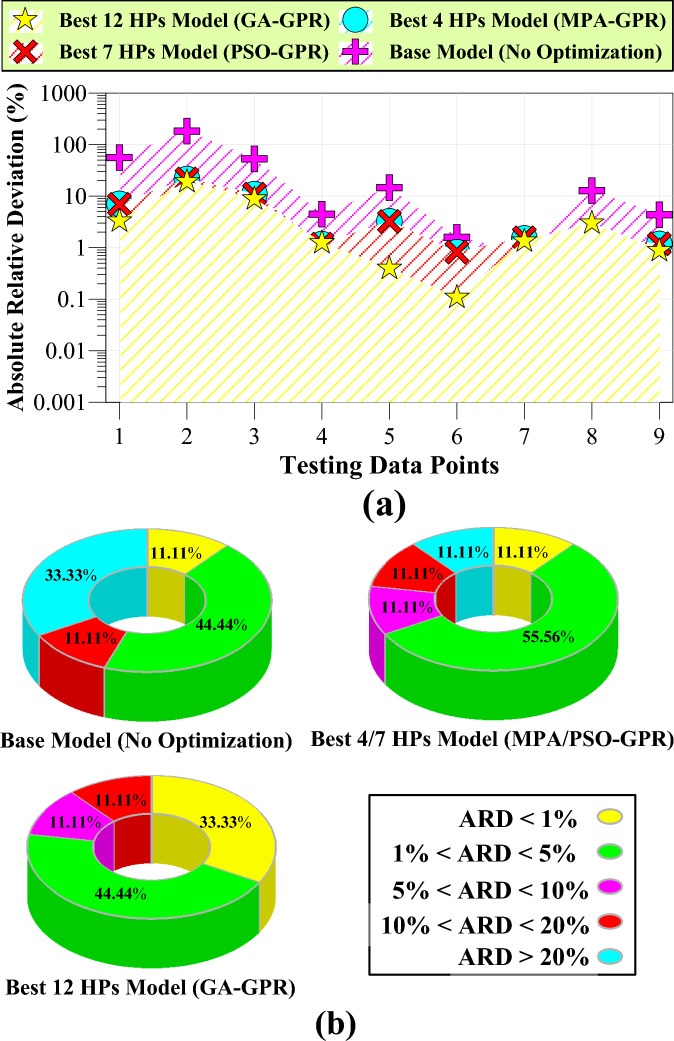
ARD of testing data points for various models.

Figure 14a presents the absolute relative deviation of the outputs of MPA-GPR, PSO-GPR, GA-GPR, and the base models from the actual values in the testing phase. Analyzing these values, we can observe distinct differences in the performance of the models. The MPA-GPR and PSO-GPR models exhibit similar ARDs, ranging from approximately 0.8% to 21.6%. These models demonstrate moderate accuracy in predicting DV values, with some variations in performance across different data points. On the other hand, the GA-GPR model demonstrates significantly lower ARDs, ranging from approximately 0.1% to 19%. This indicates that the genetic algorithm-based model provides more accurate predictions compared to the MPA-GPR and PSO-GPR models. The GA-GPR model's ability to optimize all 12 parameters results in improved performance and better capturing of the underlying patterns in the data. In contrast, the default (base) model shows much higher ARDs, ranging from approximately 0.8% to 183%. These large deviations suggest that the base model is inadequate in accurately predicting the viscosity values.

Figure 14b presents a more detailed analysis of the absolute relative deviation of different models. The figure provides additional insights into the performance of each model by focusing on specific ranges of ARD values. The basic model exhibits a relatively high ARD, with approximately 33% of the testing data recording ARD values above 20%. This indicates that the basic model struggles to accurately predict viscosity values, particularly for a significant portion of the dataset. In contrast, both the PSO-GPR and MPA-GPR models show improved performance, with only about 11% of the data recording ARD values above 20%. This suggests that these models achieve better accuracy compared to the basic model, as they have a smaller percentage of predictions with large deviations from the actual values. The most notable improvement is observed in the GPR model based on the genetic algorithm (GA-GPR). Interestingly, none of the data records an ARD above 20% for this model, indicating its ability to provide highly accurate predictions across the testing dataset. Furthermore, the analysis reveals that approximately 11% of the data for all models fall within an ARD range of 10 to 20%. This suggests a similar level of performance in capturing viscosity values within this range for all models. One noteworthy point in Fig. 14b is the data with a small error (ARD < 1%). The GA-GPR model stands out in this aspect, with approximately 33% of the data falling into this category. In contrast, the PSO-GPR, MPA-GPR, and base models have a significantly lower percentage (around 11%) of data with such a small error.

**Fig. 15:**
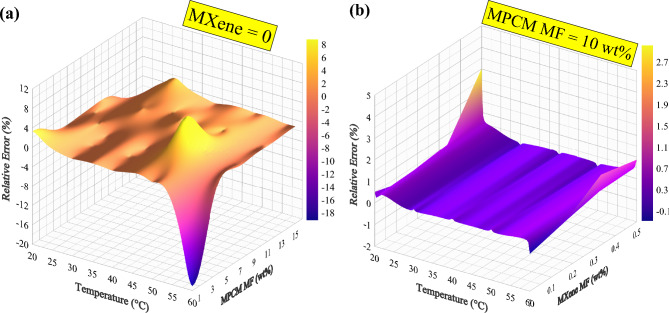
Relative error of GA-GPR outputs in terms of **(a)** T and MPCM MF for MXene = 0 and (**b**) T and MXene MF for MPCM MF = 10%.

Figure 15 provides insight into the behavior of the GA-GPR model by illustrating the relative error of its outputs in relation to the inputs. Analyzing this figure provides insights into the performance of the model under different conditions. Specifically, Fig. 15a focuses on a special case where the MXene nanomaterial is absent from the solution. Examining the relationship between temperature and RE, we can observe varying trends. Considering MPCM MF = 0 wt%, at 20 °C, the GA-GPR model produces a positive RE of 3.28%. As the temperature increases to 30 °C, the RE decreases significantly to a minimal value of 0.02%, indicating a much closer approximation to the actual values. However, at 40 °C and 50 °C, the RE remains close to zero, suggesting a relatively accurate prediction. Interestingly, at 60 °C, the model exhibits a negative RE of − 19.09%, indicating an overestimation of the viscosity values. When the MPCM dosage is increased to 5 wt%, the highest relative error is observed at 50 °C. The model exhibits a relative error of 8.8% at this temperature, indicating the most significant positive deviation from the actual values. This suggests that the GA-GPR model underestimates viscosity values in this particular range. However, when the MPCM mass fraction is set to 10 wt%, the relative error remains around or below 1%. This indicates a higher level of accuracy and suggests that the model performs well in predicting viscosity values in this dosage range. Interestingly, when the MPCM mass fraction is further increased to 15 wt%, a reasonable percentage error is only observed at low temperatures, specifically between 20 and 30 °C. Figure 15b highlights the relative error of the superior model under a specific condition where a constant dose of MPCM (10 wt%) is present. In this scenario, the model error remains within acceptable limits for most problem conditions, with a relative error below 1%. However, a notable exception occurs when the lowest temperature (20 °C) is combined with the maximum MF of MXene (0.5 wt%). In this case, the model shows a RE = 3%, indicating a slightly larger deviation from the experimental values.

**Fig. 16:**
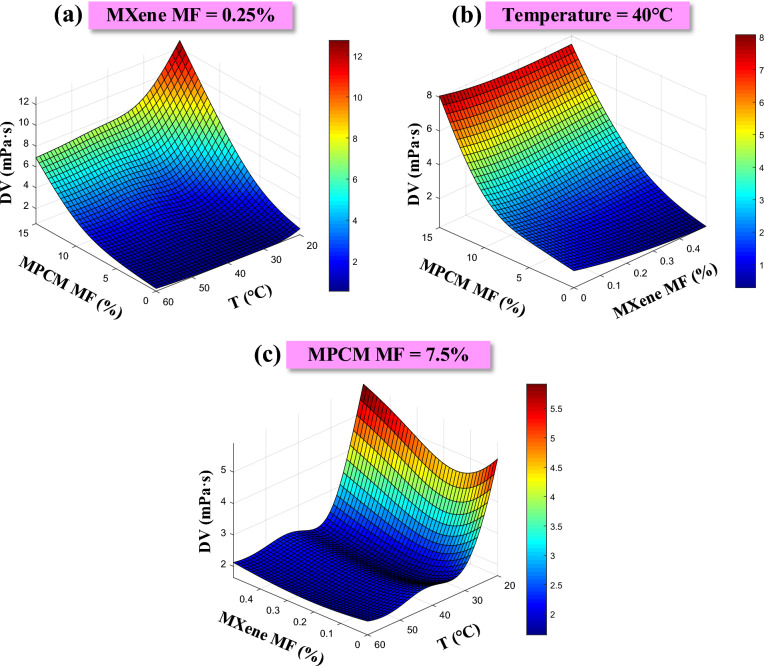
Dynamic viscosity outputs of GA-GPR model for suspension containing MPCM and MXene under the influence of MPCM MF, MXene MF and Temperature.

Figure 16 presents the dynamic viscosity variation of the suspension that comprises microencapsulated PCM and MXene, as influenced by the MPCM MF, MXene MF, and temperature. These insights are derived from the GA-GPR model. According to the figure, a decrease in temperature leads to an increase in the DV of suspension. The severity of this phenomenon can be attributed to the behavior of the suspending medium and the particles within it. At lower temperatures, the kinetic energy of the particles decreases, causing them to move more slowly. As a result, the particles tend to interact and come closer together. This increased interaction and reduced particle movement lead to stronger intermolecular forces and tighter packing of particles within the suspension. Increasing the MPCM MF in the base fluid raises the effect of temperature on viscosity.

On the other hand, increasing the concentration of MPCM causes a substantial increase in viscosity. As the concentration of MPCM increases, more particles are dispersed throughout the suspension. These particles have a certain size and shape, which affects their interaction with the suspending medium and other particles. When the concentration of MPCM is low, the particles are relatively dispersed and spaced apart within the suspension. This allows the suspending medium to flow more freely between the particles, resulting in lower resistance to flow and lower dynamic viscosity. However, as the concentration of MPCM increases, the particles become more closely packed together. This reduces the space available for the suspending medium to flow between the particles, leading to increased frictional forces and resistance to flow. Consequently, the dynamic viscosity of the suspension increases significantly. According to the figure, the effect of MXene NMs on viscosity is relatively tiny. However, it is essential to note that MXene have a noteworthy influence on other thermophysical properties of the suspension, such as thermal conductivity.

## Conclusion

This study uses Gaussian process regression to accurately predict the dynamic viscosity of suspensions containing microencapsulated PCM and MXene nanomaterials. Twelve hyperparameters of GPR are analyzed separately and classified into three groups based on their importance. Three metaheuristic algorithms, namely GA, PSO, and MPA, are employed to optimize these HPs. The developed models provide engineers with a deep understanding of dynamic viscosity in pure and nano-enhanced MPCM suspensions. This research offers a tool for optimizing machine learning methods with numerous hyperparameters. These advancements significantly affect efficiency in various industries, from thermal energy storage to thermal management systems. Moreover, the models reduce experimental and computational costs, providing cost-effective and efficient solutions. The most important findings of this study are as follows:

- Twelve HPs of GPR were investigated. Among these, the covariance function, basis function, standardization, and sigma were found to have the most significant impact on the GPR model. These HPs were optimized as part of the first group.
- The second group of HPs incorporated the four primary HPs along with supplementary parameters that had a minimal impact (less than 1%) on the model's accuracy. These secondary HPs encompassed the fit method, predict method, and optimizer.
- Altering the values of certain HPs (active set method, active set size, distance method, computation method, and regularization) did not impact the accuracy of the default model.
- To explore the interactions between HPs, the third group was created, encompassing all of the HPs.
- The GA, PSO, and MPA algorithms achieved similar performance results when optimizing the first group of HPs. The R, R^2^, and I_A_ values (0.998303, 0.996608, and 0.999135) indicated reasonable performance for all three algorithms. However, when considering errors, the MPA-GPR model showed a slightly lower MAPE on the testing dataset.
- The models optimized with seven HPs in the second group, using various algorithms, showed similar accuracy to those in the first group. However, the PSO-based model outperformed the other two models in key evaluation metrics such as MSE, R, R^2^, and I_A_.
- The optimized models, involving 12 different HPs, revealed that the genetic algorithm successfully identified a unique combination of HPs. The GA-GPR model exhibited excellent compliance with the targets, with high values of R (0.999224) and R^2^ (0.998449) achieved on the testing data.
- The statistical criteria, regression charts, violin plots, and absolute relative deviation charts revealed the significant superiority of the optimized model from the third group (12 HPs) compared to other HP groups. This highlights the importance of mutual interactions among HPs.

This study presents a valuable tool for engineers and decision-makers in industries reliant on microencapsulated phase change materials. Suspensions containing MCPMs and their characteristics are fundamental for optimizing processes and product performance in TES systems, construction, thermal management, and electronics. The proposed methodology of hyperparameter optimization for machine learning models empowers industries to achieve significant improvements in these fields. This translates into concrete advantages, including reduced laboratory experimentation costs, enhanced decision-making in material selection and process design, ultimately leading to optimized processes across a diverse range of applications. The efficacy of this approach is further substantiated by the success of metaheuristic algorithms in identifying optimal hyperparameter combinations, resulting in highly accurate models.

## Data Availability

The datasets used and analysed during the current study available from the corresponding author on reasonable request.
